# Characterization of leaf surface phenotypes based on light interaction

**DOI:** 10.1186/s13007-023-01004-2

**Published:** 2023-03-18

**Authors:** Reisha D. Peters, Scott D. Noble

**Affiliations:** 1 Chemical and Biological Engineering, University of Saskatchewan, 57 Campus Drive, Saskatoon, SK S7N 5A9 Canada; 2 Mechanical Engineering, University of Saskatchewan, 57 Campus Drive, Saskatoon, SK S7N 5A9 Canada

**Keywords:** Polarization, Leaves, Light, Reflectance, Pubescence, Wax, Surface roughness

## Abstract

**Background:**

Leaf surface phenotypes can indicate plant health and relate to a plant’s adaptations to environmental stresses. Identifying these phenotypes using non-invasive techniques can assist in high-throughput phenotyping and can improve decision making in plant breeding. Identification of these surface phenotypes can also assist in stress identification. Incorporating surface phenotypes into leaf optical modelling can lead to improved biochemical parameter retrieval and species identification.

**Results:**

In this paper, leaf surface phenotypes are characterized for 349 leaf samples based on polarized light reflectance measured at Brewster’s Angle, and microscopic observation. Four main leaf surface phenotypes (glossy wax, glaucous wax, high trichome density, and glabrous) were identified for the leaf samples. The microscopic and visual observations of the phenotypes were used as ground truth for comparison with the spectral classification. In addition to surface classification, the microscope images were used to assess cell size, shape, and cell cap aspect ratios; these surface attributes were not found to correlate significantly with spectral measurements obtained in this study. Using a quadratic discriminant analysis function, a series of 10,000 classifications were run with the data randomly split between training and testing datasets, with 150 and 199 samples, respectively. The average correct classification rate was 72.9% with a worst-case classification of 60.3%.

**Conclusions:**

Leaf surface phenotypes were successfully correlated with spectral measurements that can be obtained remotely. Remote identification of these surface phenotypes will improve leaf optical modelling and biochemical parameter estimations. Phenotyping of leaf surfaces can inform plant breeding decisions and assist with plant health monitoring.

## Background

Using spectral measurements to estimate leaf biochemical properties has been a developing field for a number of decades with early work relating leaf structure and reflectance [1, 2]. Advances in this field have led to models and indices that can estimate the biochemical properties of leaves using reflectance and transmittance spectra [3–7]. Beyond the biochemical properties of leaves, reflectance and transmittance spectra are affected by illumination angle and leaf surface properties. The effects of illumination angle have been studied and modeled by combining the PROSPECT model with other features (BRDF [8] or the COSINE model [9]) but the effects of the leaf surface have not been investigated extensively in the context of modelling using spectral measurements.

Leaf surface characteristics are an important factor when considering light reflectance and transmittance as they can affect the absorbance of the sample and the scattering of light at the leaf surface [10]. This latter feature is particularly important when considering illumination at angles other than nadir [11]. Specular reflection occurs at the leaf-cuticle interface when light is specularly reflected before it encounters any biochemical constituents within the leaf [8, 11]. This specular light is partially polarized based on the surface roughness, angle of illumination, and index of refraction of the leaf surface [8, 11]. Conversely, diffuse light is reflected after being scattered within the interior of the leaf and is unpolarized [8, 11, 12].

Studies have been conducted to investigate the effects of leaf surface characteristics on light polarization [12–14] with an important finding that the optical characteristics of a leaf surface have effects on both the measured specular and diffuse components of reflectance [14]. This may be due to the non-uniformity of specular reflectance over multiple wavelengths as the index of refraction of the leaf cuticle tends to increase towards shorter wavelengths [5, 9]. Changes in the specular reflectance necessarily cause changes in the diffuse component at a given wavelength. Other findings have indicated that that the specular reflectance is independent of pigment content in the leaves at Brewster’s angle [11, 15]. However, the scope of these previous studies was limited to individual wavelength comparisons or a series of 5 wavelengths in the visible region and the uneven nature of leaf surfaces is not well suited to exact discrimination between the diffuse and specular components.

Research to evaluate and model polarization from plant canopies has assessed illumination angle [16] and canopy-level phenological traits such as flowering or a general glossy appearance of the leaves [17–19]. These studies investigated the polarization factor through a wider wavelength range and were conducted to better understand the scattering of vegetation covers but did not relate the polarization directly to the surface features at the cellular level.

Researchers have expressed interest in combining leaf surface characteristic modelling with biochemical modelling but a shortage of leaf surface characteristic data that are correlated with specific spectral data has prevented the furthering of this research [20]. Although polarization reflectance data exist for a variety of species at various angles [8, 15, 16], measured data in regards to the physical structure and characteristics (waxiness or trichome density) of the corresponding leaf surface is missing. Cell size and surface undulation, trichomes, and epicuticular wax can affect the polarization of a leaf surface. Studies on the structure and function of these three features and how they relate to plant robustness, environmental interaction, and pesticide wetting are extensive; however, the effect of these structures on light and leaf optical properties has not been presented in the range or with the resolution required for integration with current leaf models.

Additional work by Boize describing the structure of surface features has further categorized leaf surface roughness into three subsections [21]. The macroscopic roughness considers features such as trichomes and protruding veins (~ 200–1000 µm), the microscopic roughness considers the cell size and arrangement (~ 10–200 µm), and the ultra-microscopic roughness considers the size and shape of the epicuticular wax system (~ 1–10 µm) [21]. The macroscopic and microscopic roughness features are much larger than the wavelengths in the UV-Visible-NIR region of the spectrum and will affect the direction of light reflection based on variable local angle of incidence as the surface may not be optically smooth. The ultra-microscopic features approach the NIR wavelengths which may result in unique light interactions in different regions of the spectrum. Although the epicuticular waxes are classified as ultra-microscopic, their effects are still noticeable at the microscopic level as the waxes have the potential to form large structures on a scale similar to the inter-cellular grooves [21, 22].

The size and undulation of epidermal cells determine the local surface orientation; this is related to the surface roughness of the leaf and results in changes in local angle of incidence affecting the specular light that is reflected at the leaf surface. Epidermal cells can have diverse shapes and sizes with a range of length-to-width ratios [21] and margins with varying degrees of undulation [23]. The height of the epidermal cells in relation to their width and length and the undulation of the margin will also affect the local surface orientation. These features have not been extensively considered when studying polarization as related to leaf surface phenotypes but a link between large features that approximate a plane surface (possibly large, flat epidermal cells) have been noted as producing more specular light [14]. Previous studies have generalized that older leaves appear to have a rougher cellular surface than young leaves [24] and would therefore produce a more diffuse reflectance. This study focused primarily on the microscopic roughness scale (~ 10–200 µm).

Trichome size, shape, and density can play a major role in the direction of light scattering [8, 14, 25]. Trichome shape can be diverse ranging from short and pointed to long rounded and hooked at the end with a single or multiple branches [26]. At the most basic classification, trichomes are often described as either glandular or non-glandular. A glandular trichome is one which is capable of accumulating chemicals such as phytotoxic oils that can potentially be useful in deterring herbivores, guiding pollinators, or affecting photosynthesis [27]. The density of hairs on leaves is variable across the leaf surface with densities often increasing on the veins [21].

Epicuticular waxes can produce absorption features unique from the biochemical constituents beyond the leaf cuticle [10]. These waxes can also produce structural features that affect light polarization similarly to the cell size and trichomes. The epicuticular wax generally lies at the air-leaf interface at the top of the cuticle and has been hypothesized to minimize mechanical damage, inhibit insect attack, and protect from excess UV radiation [28]. The epicuticular wax can also improve drought tolerance by inhibiting cuticular transpiration [28]. The small structural features of the waxes have the potential to scatter shorter wavelengths more effectively than longer wavelengths [15], a phenomenon that is not seen with the cellular roughness or trichome features. These waxes can be very diverse both at the visual level and structural level. Some waxes appear glossy and shiny while others appear glaucous and produce a waxy bloom [22, 29]. At the ultra-microscopic level, the structures of these waxes have been studied extensively and classified by observing over 13000 species. Barthlott et al. classified 23 types of waxes that include smooth layers, platelets, and rodlets that can be correlated back to some of the visual representations of these waxes at the leaf-level [22].

The work described in this paper attempts to link leaf surface properties (surface roughness, trichomes, and epicuticular waxes) to spectral measurements for the purpose of integrating these features into future modelling. Quantitative comparisons between measured roughness parameters and qualitative classification of generalized surface phenotypes (e.g. waxy or not waxy) are investigated. Polarized light reflectance is used to investigate the effects of leaf surface phenotypes in this preliminary study towards their incorporation into spectral modelling. For the purposes of this study, the observations of the physical characteristics were limited to the macroscopic and microscope roughness as evaluated by an optical microscope and visual examination. The glaucous or glossy wax presentation was noted but electron microscope images were not examined. Using optical microscopic assessment, the roughness of the surface cells, trichomes shape and density, and macroscopic structure of the waxes were analyzed and compared to spectral measurements of leaf surfaces. These results were used to create a classification protocol for predicting leaf surface phenotypes from polarized spectral measurements.

## Methods

**Fig. 1:**
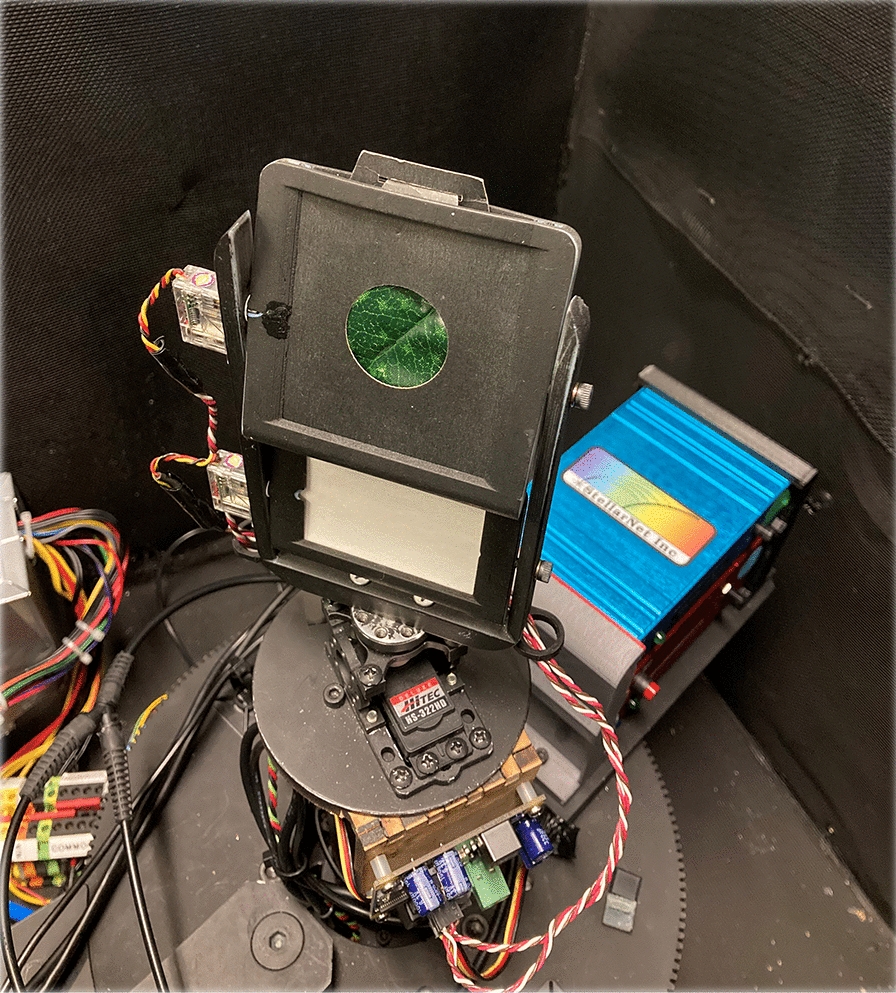
Leaf holder used to ensure the same part of the leaf is assessed in every stage of the data collection

Leaf samples were selected from indoor and outdoor sources, with the objective of developing a dataset representing a wide range of trichome density, wax expression, cellular roughness, and pigmentation. Leaves were excised from the plants in a greenhouse or outdoors and stored in plastic bags in a dark cooler full of ice [30]. Air was blown into the plastic bags by mouth to increase the humidity (as described in the spectronomics protocol referenced in [30]) and prevent the adaxial surface of the leaf from contacting the plastic bag. These leaves were analyzed with a microscope and their spectral measurements were obtained within four hours of removal from their plants. All measurements for a single sample were collected within 30 min from start to finish (with replacement in the dark cooler between measurements if necessary) to reduce changes in the leaf surface structure during assessment. To ensure the same location on each leaf was analyzed microscopically and spectrally, the leaves were placed in a sample holder that centered the area of interest to be examined for each measurement as shown in Fig. 1 [31]. In total, 349 leaves and associated measurements were included in the dataset, representing 59 different species. These are summarized in Table 1. A large portion of these leaves are from the LOTUS dataset [32].

**Table 1:** Sample species and number used in this study

Species	# of samples	Species	# of samples
*Abelmoschus esculentus* (L.) Moench	3	*Ligularia sibirica* (L.) Cass	1
*Acer negundo* L	4	*Malus* sp. Mill	5
*Amelanchier alnifolia* (Nutt.) Nutt	5	*Nicotiana tabacum* L	4
*Anthurium* Schott	1	*Ocimum basilicum* L	2
*Begonia* sp. L	2	*Parthenocissus quinquefolia* (L.)^†^	4
*Beta vulgaris* L	2	*Penstemon barbatus* (Cav.) Roth	1
*Betula* L	2	*Petunia* sp. Juss	1
*Brassica napus* L	83	*Phaseolus vulgaris* L	45
*Brassica oleracea* L	2	*Populus tremuloides* Michs	1
*Capsicum annuum* L	11	*Prunus* sp. L	13
*Capsicum baccatum* L	1	*Quercus* sp. L	10
*Catharanthus* sp. (L.) G.Don	1	*Rubus idaeus* L.^‡^	3
*Celosia* sp. L	1	*Rumex acetosa* L	1
*Citrus limon* (L.) Osbeck Modernism	2	*Salvia* sp. L	2
*Coleus scutellarioides* (L.) Benth	1	*Sambucus racemosa* L	1
*Coriandrum sativum* L	2	*Solanum lycopersicum* L	9
*Cornus* sp. L	5	*Solanum tuberosum* L	6
*Crassula ovata* (Miller) Druce (1917)	10	*Spiraea* sp. L	1
*Dianthus barbatus* L	1	*Stachys byzantine* K.Koch	1
*Eucalyptus regnans* F.Muell	1	*Streptocarpus* sect. *Saintpaulia* H.Wendl	2
*Fragaria x ananassa* Duchesne	5	*Symphoricarpos* sp. Duhamel 1755	1
*Fraxinus pennsylvanica* Marshall	5	*Taraxacum* sp. F. H. Wigg	3
*Geranium* sp. L	2	*Tradescantia* sp. L	8
*Glycine max* (L.) Merr	6	*Tropaeolum* sp. L	2
*Helianthus annuus* L	4	*Typha* sp. L	2
*Helianthus tuberosus* L	6	*Ulmus americana* L	4
*Hosta* sp. Tratt.^§^	2	*Verbena* sp. L	1
*Hypoestes phyllostachya* Baker, 1887	2	*Vitis vinifera* L	3
*Lactuca sativa* L	5	*Zea mays* L	7
*Lathyrus odoratus* L	3	Unknown	26

^§^conserved name, not Jacq. (syn of *Cornutia* in Lamiaceae) nor Vell. Ex Pfeiff (Primulaceae)

^†^*Parthenocissus quinquefolia* (L.) Planch

^‡^*Rubus idaeus* L. 1753 not Blanco 1837 nor Vell. 1829 nor Pursh 1814 nor Thunb. 1784

### Microscope analysis and surface reconstruction

A polarizing metallurgical microscope (ME580TA-PZ-2L-18M3, AmScope) was used to assess the leaf surfaces. Due to the rough surface of the leaf samples, a single microscope image did not produce an in-focus image of the entire window. To obtain both depth information and fully focused images, a focus stacking technique was employed. Depending on the topography of the leaf surface, between 10 and 50 images were collected for each sample at different microscope stage heights. These series of images were obtained at 100 × and 500 × magnification to highlight the macroscopic and microscopic surface roughness features respectively. Images were taken at stage heights every 0.005 mm for 100 × magnification and every 0.001 mm for 500 × magnification.

Post processing of these images involved stitching the series of images together using a focus stacking technique that assessed the gradient of each pixel in all images and determined the highest-gradient image to be “in focus” for each pixel. A symmetric 10 × 10 median filter was applied to the 1842 × 2456 image to remove noise and then a 15 × 15 Gaussian filter was applied to reduce small local variations and better capture the shape of a single cell. This generated a single, in-focus, composite image. Each pixel was then mapped back to three-dimensional space based on the in-focus height that was determined during stitching. By draping the stitched image of the leaf onto the three-dimensional plot, the leaf surface roughness was reconstructed. This allowed for quantitative assessment of cellular roughness and trichome height, shape, and density and provided visualization of all features. The program for stitching and 3D reconstruction of these images was developed specifically for this project using Matlab [33].

**Fig. 2:**
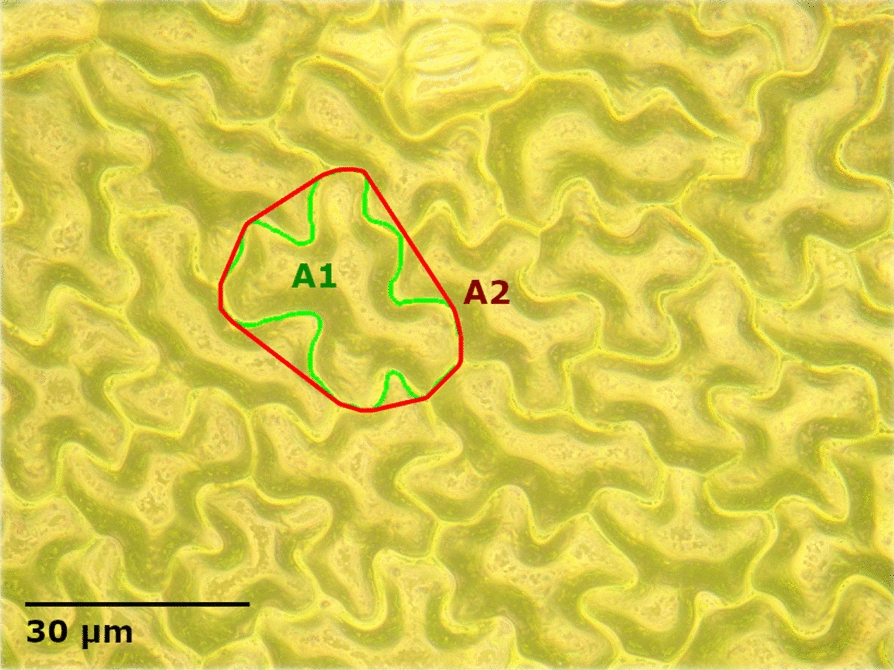
Microscope image of a black bean (*Phaseolus vulgaris* L.) leaf at 500 × magnification showing the user traced cell (A1) and computed convex hull (A2) used in cell size and margin undulation calculations

Quantifying the cellular roughness in a single numerical parameter is difficult as variations in cellular size, shape, and aspect ratio can all affect the apparent roughness of the surface. To capture this characteristic, a number of metrics were assessed. The cellular size, the degree of undulation of the cellular margin (margin undulation), and cell cap aspect ratio were evaluated using the composite image and the 3D reconstructions of the 500 × magnification images. To determine the cell size, margin undulation, and cell cap aspect ratio, a single cell was manually traced on the composite 500 × magnification image (as shown in Fig. 2). Cell size was determined based on the number of pixels within the traced area of a single cell and converted to square micrometers based on measurements of a microscope calibration slide. The margin undulation was determined as the ratio between the cell area and the area of a computed convex hull around the traced cell as show in Eq. 1 using the areas A_1_ and A_2_ traced in Fig. 2. The margin undulation will be a value between 0 and 1 where 1 represents a completely round cell.

1 $$Margin~\,Undulation = \frac{{A_{1} }}{{A_{2} }}$$

**Fig. 3:**
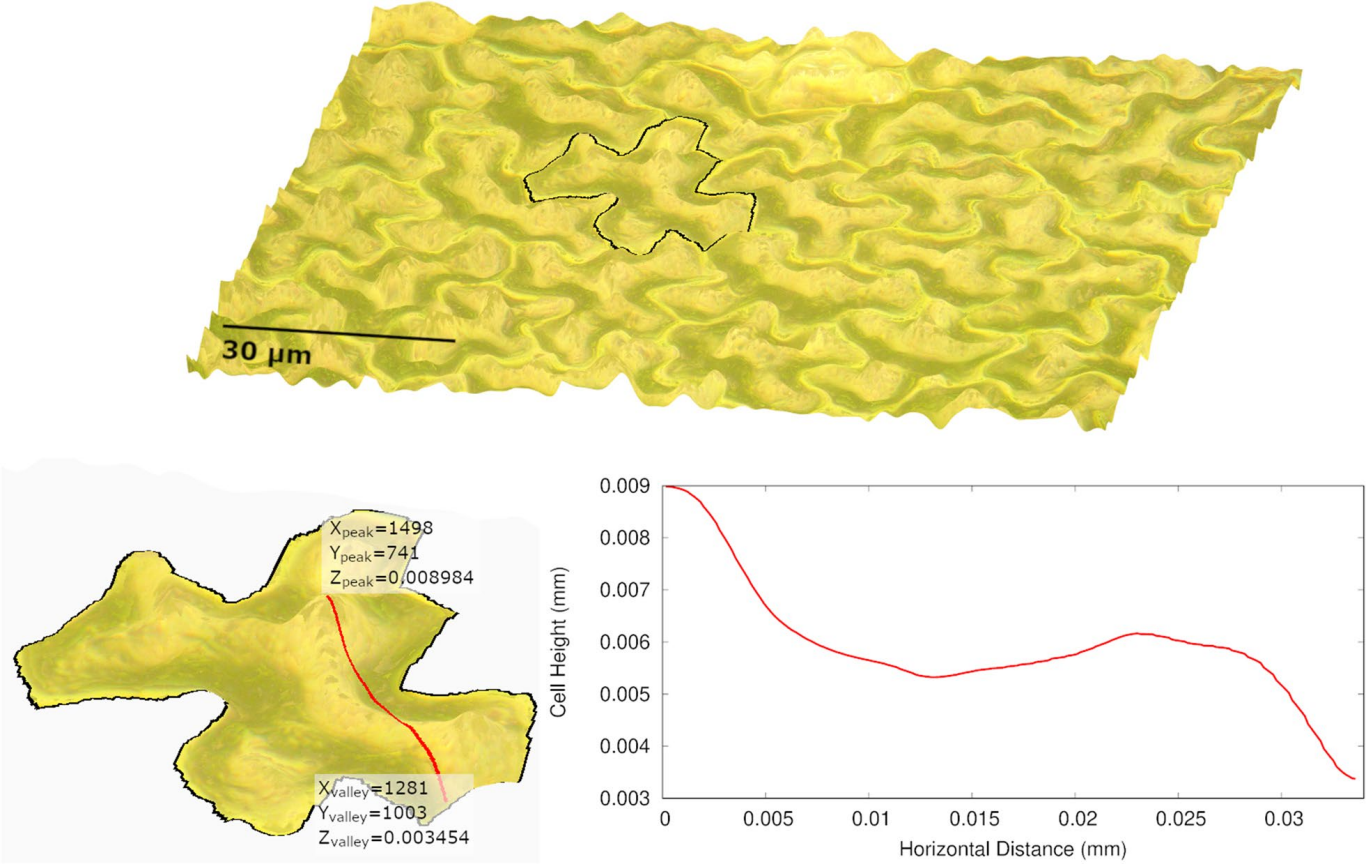
3D reconstruction of a black bean (*Phaseolus vulgaris* L.) leaf showing possible points used for cell cap aspect ratio calculations. X and Y axis values are pixels (1 pixel = 0.098 µm) and Z axis values are in millimeters

The cell cap aspect ratio was calculated using the values on the 3D reconstruction that were within the traced cell area. Cell cap aspect ratio divides the width of a cell by the height of the cell. The difference between the maximum and minimum heights from within the cell area were taken as the cell height (in micrometers) and the cell width was determined based on the coordinate locations of those maximum and minimum heights. The cell width was calculated as twice the Pythagorean distance between the two points (in micrometers) and the cell cap aspect ratio was recorded as the ratio of width to height. Figure 3 shows example values for the cell cap aspect ratio calculations and Eq. 2 describes how these values are used.

2 $$Cell\,Cap\,Aspect\,Ratio = \,\frac{{2c\sqrt {\left( {x_{peak} - x_{valley} } \right)^{2} + \,\left( {y_{peak} - y_{valley} } \right)^{2} } }}{{\left( {z_{peak} - z_{valley} } \right)}}$$

where *x*_*peak*_, *y*_*peak*_, and *z*_*peak*_ correspond to the highest point, *x*_*valley*_, *y*_*valley*_, and *z*_*valley*_ correspond to the lowest point, and $c$ is a scaling factor equal to $9.8\times {10}^{-5}$ $mm/pixel$. This scaling factor is used to convert pixel number to millimeters using the calibration slide. For the example shown in Fig. 3, the cell cap aspect ratio is 12.1.

Five measurements (cell tracings) were recorded for each sample and an average value of the five repetitions was used for investigating correlations with spectral measurements. For 12 of the 349 samples, it was difficult to obtain cellular measurements due to very high amounts of wax deposits or trichomes covering the epidermal cells. These samples were not used when determining correlations between cellular surface roughness and spectral measurements but were used in surface phenotype identification.

### Spectral analysis

Light that is reflected from an optically smooth surface is partially polarized perpendicular to the plane of incidence based on the illumination angle and refractive index. For samples that are not optically smooth (e.g. leaves), the degree of polarization is also dependent on the surface characteristics of the sample. Light is either reflected at the air-leaf interface or transmitted to the interior of the leaf where subsequent absorption, reflection, or transmittance occurs. Light that does not scatter specularly from the surface is scattered diffusely within the leaf (as reflectance or transmittance) and is depolarized. Spectral measurements were obtained between 400 and 1700 nm using the GoSPo goniospectropolarimeter [31] with illumination and sensor at Brewster’s Angle (approximately 55° from nadir using a refractive index of 1.45). A wire grid polarizing filter was placed between the sample and sensor (Edmund Optics, Barrington, NJ, USA). Reflectance factor spectra were collected with this polarizer in different orientations between 0° (parallel polarization to the incident plane) and 90° (perpendicular polarization to the incident plane) in 5° increments. These measurements were corrected to the total light as measured directly through the polarizing filter. The polarized bidirectional reflectance factor was estimated using Eq. 3 [14].

3 $$R_{Q} = \frac{{R_{max} - R_{min} }}{2.0}$$

where *R*_*Q*_ is the polarized bidirectional reflectance factor, *R*_*max*_ is the bidirectional reflectance factor in the perpendicular polarizer orientation, and *R*_*min*_ is the bidirectional reflectance factor in the parallel polarizer orientation. *R*_*min*_ should also be equal to the diffuse reflectance and ${R}_{Q}$ is equal to the specular reflectance. The sum of *R*_*min*_ and *R*_*Q*_ should equal the total bidirectional reflectance factor. *R* [14].

4 $$R = \frac{{R_{max} + R_{min} }}{2.0} = R_{Q} + R_{min}$$

When collecting these polarized reflectance data, there was a thin film on the polarizer that caused spectral interference. Undulations in the specularly reflected light that were due to this interference were mitigated by using the average reflectance factor values between 500 and 900 nm for the analysis. The averaging allowed for the increases and decreases in the specular light due to the interference to cancel out over the range of interest.

For each leaf, nineteen reflectance factor scans were taken between 400 and 1700 nm at 5° increments of the polarizer’s orientation. Many scans were necessary to capture the maximum and minimum spectra as the surface of the leaf is not always perfectly flat and aligned at 55° illumination. For each scan, a dark reference spectrum was removed and the scan was normalized to the total transmittance through the polarizing filter (taken with source and sensor aligned). Two parameters were then calculated from each series of scans using the maximum and minimum spectra. The average *R*_*Q*_ value (*R*_*Qav*_) was determined as the average of values between 500 and 900 nm and the diffuse ratio (*DIFF*_*R*_) was determined as the reflectance factor ratio between 765 and 680 nm in the diffuse spectrum (minimum spectrum or *R*_*min*_). The *R*_*Qav*_ range was chosen to maximize the number of data points in the least noisy portions of the spectra, and the *DIFF*_*R*_ wavelengths were chose to span the red edge of the reflectance curve (where the leaf pigments no longer play a large role in the reflectance).

## Results and discussion

In this section, the processing of spectral measurements and the observations of the effects of three leaf surface phenotypes will be discussed. The three surface phenotypes include the cellular roughness on the surface of the leaf, the presence and types of waxes on the surface of the leaf, and the trichome shape, size, and density on the surface of the leaf. Following the analysis of the effects of each phenotype on light interaction with the leaf, a method for classifying the leaf surface phenotype using polarized spectral data will be presented.

### Specular measurements

**Fig. 4:**
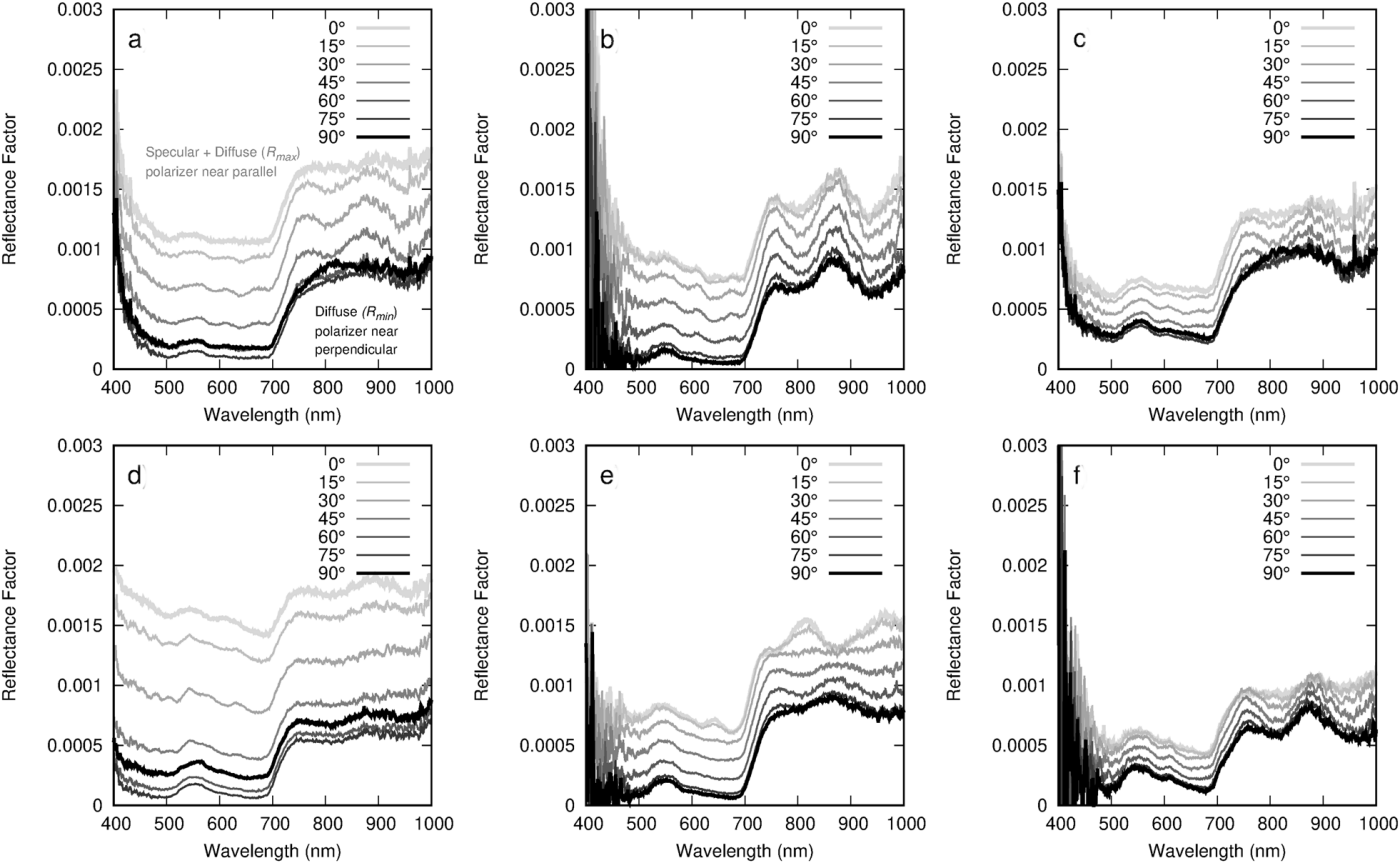
Reflectance factor with different polarizer orientations between parallel and perpendicular for **a** Virginia creeper (*Parthenocissus quinquefolia* (L.) Planch.) **b** Black bean (*Phaseolus vulgaris* L.) **c** Okra (*Abelmoschus esculentus* (L.) Moench) **d** Begonia (*Begonia* sp. L.) **e** Lemon (*Citrus limon* (L.) Osbeck) **f** Soy (*Glycine max* (L.) Merr.). These leaves correspond with the images in Fig. 5

A selection of scans obtained from six leaf sample are shown in Fig. 4. The diffuse reflectance is the minimum reflectance factor spectra and *R*_*Qav*_ is calculated using the difference between the maximum and minimum spectra. Theoretically, the minimum reflectance should be obtained with the polarizer parallel to the plane of incidence. Due to the potential for leaf surface angle variation, *R*_*min*_ and *R*_*max*_ were determined based on the lowest and highest measured reflectance respectively, instead of the reflectance measured at the defined parallel and perpendicular polarizer orientations. This is seen in Fig. 4 where the spectra at 90$^\circ$ is not always the minimum.

In Fig. 4, there is a shape difference between the maximum and minimum reflectance factor spectra that results from the differences in specularly and diffusely reflected light. The maximum reflectance contains both specularly and diffusely reflected light, but the minimum reflectance only contains diffusely reflected light. The portion that is specular light appears more white than green as this light does not enter the leaf and interact with biochemical components. The specular reflectance in the visible region of the spectra causes the maximum reflectance factor spectra to appear flatter than in the diffuse reflectance and these spectra are partially polarized. The diffuse reflectance represents light that enters the leaf and is reemitted after encountering one or more interfaces inside the leaf. This light appears green as it has been partially absorbed by the biochemical constituents and is non-polarized. In these data, there is a thin film interference effect from the polarizer that causes undulations in the specularly reflected light. This effect is most noticeable in samples with high ${R}_{Q}$ values but is mitigated by using the average value over 500–900 nm. In Fig. 4, this effect can be seen as waves in some of the spectra but the effect is generally less prominent at ${R}_{min}$ and ${R}_{max}$. Removal of this effect has not been possible with correction with light measured through the polarizing filter as the degree of severity of the interference appears to change between samples.

### Cellular roughness

**Fig. 5:**
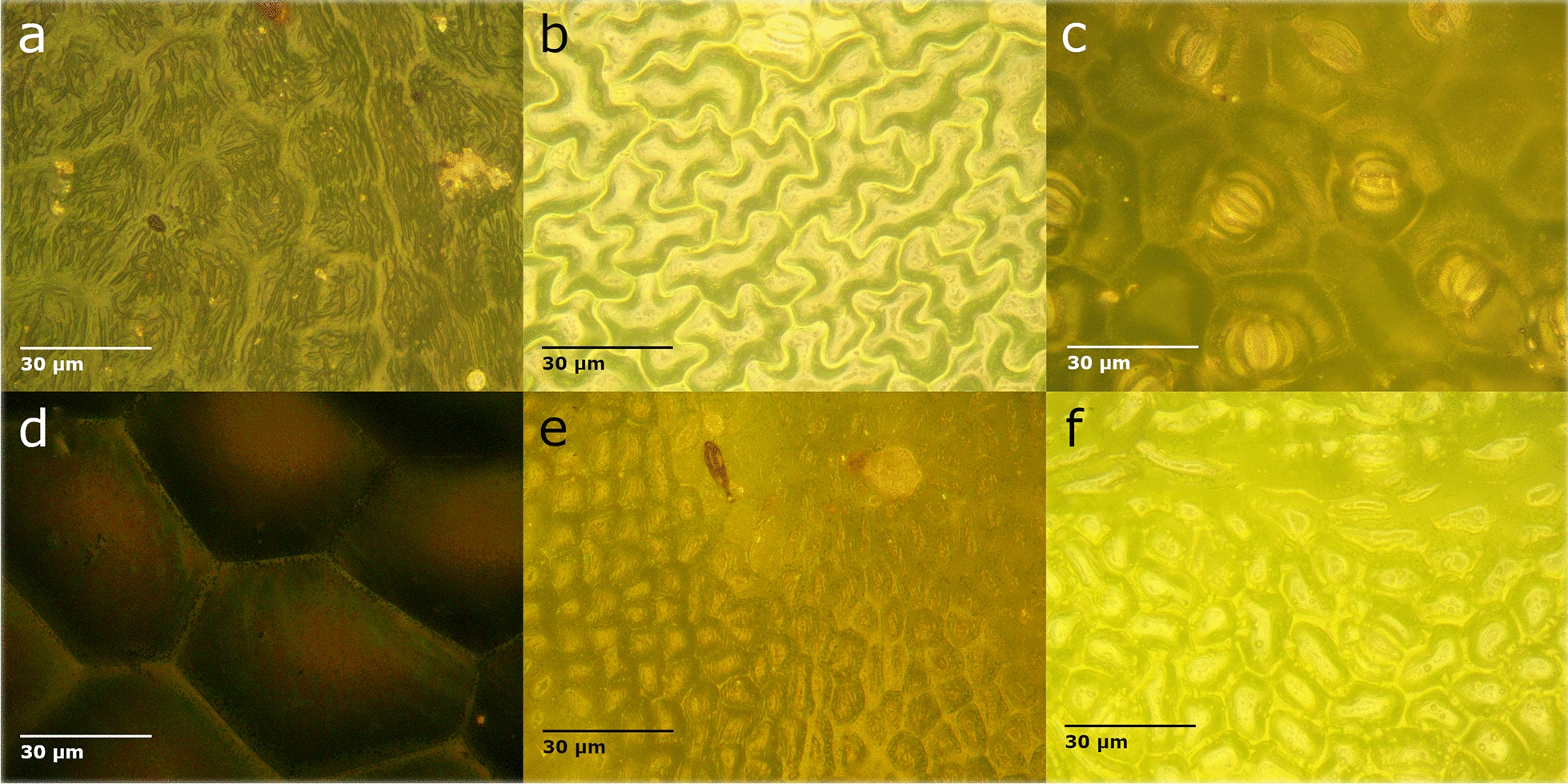
A variety of cell shapes and sizes at 500 × magnification for **a** Virginia creeper (*Parthenocissus quinquefolia* (L.) Planch.) **b** Black bean (*Phaseolus vulgaris* L.) **c** Okra (*Abelmoschus esculentus* (L.) Moench) **d** Begonia (*Begonia* sp. L.) **e** Lemon (*Citrus limon* (L.) Osbeck) **f** Soy (*Glycine max* (L.) Merr.)

Figure 5 shows a selection of images with leaf cells that range from small to large, low to high undulating edges, and low to high cell cap aspect ratios, depicted in no relative order. These samples correspond to the spectral data show in Fig. 4. The biophysical metrics for these samples are summarized in Table 2 as well as the metric obtained from spectral measurements. In this table, the margin undulation represents values up to 1 (which would represent a perfectly round cell). A larger value for cell cap aspect ratio represents a flatter surface.

**Table 2:** Summary of cellular features and spectral measurements for six microscope images shown in Fig. 5

	a. Virginia creeper	b. Black bean	c. Okra	d. Begonia	e. Lemon	f. Soy
Cell size (μm^2^)	982	1773	1497	8448	260	568
Margin undulation	0.96	0.77	0.84	0.97	0.95	0.95
Cell cap aspect ratio	21.4	17.1	30.4	18.6	9.2	9.1
*R*_*Qav*_ (× 10^4^)	4.85	3.72	2.32	6.77	3.03	1.63
*DIFF*_*R*_	6.83	13.17	4.77	9.67	11.80	5.17

**Fig. 6:**
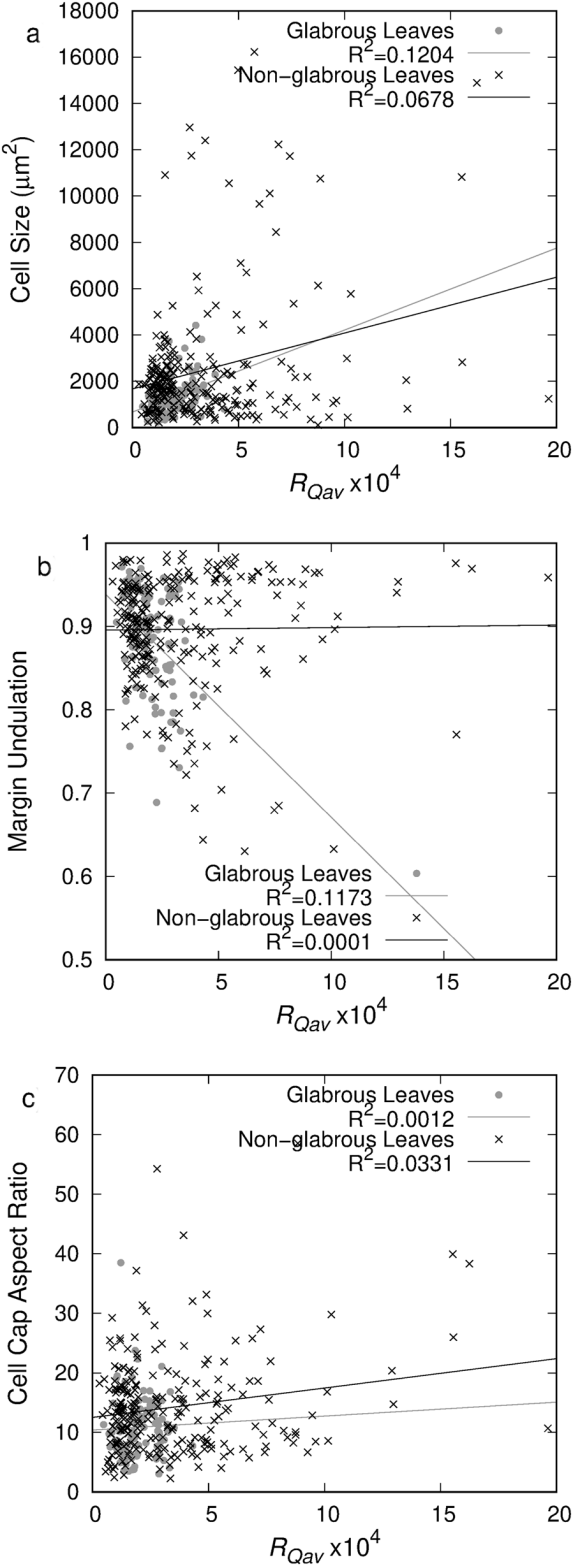
Comparison of leaf surface parameters to *R*_*Qav*_ value for **a** Cell Size **b** Margin Undulation **c** Cell Cap Aspect Ratio. Full dataset shown with black x, glabrous leaves shown with grey dot

To determine the effects of these measured cellular parameters on the spectral measurements, the cellular feature values were plotted against the *R*_*Qav*_ value for each leaf as shown in Fig. 6. In this set of figures, the cell size, margin undulation, and cell cap aspect ratio correlations are shown. Other parameters (e.g. cell length to width ratio, stomata count) were observed in some leaves, but the margin undulation and size were the only parameters to show some correlation with the measured *R*_*Qav*_. The cell cap aspect ratio correlations are included as it is a parameter currently used in some leaf models [20].

In Fig. 6, two subsets of data and two trend lines are determined. The first subset of data was the glabrous leaves which did not have a significant number of trichomes or quantity of wax observed in the microscope images. This subset is denoted by the grey dot marker and grey trend line. The second, larger subset includes all other leaves in the dataset for which microscopic observation was possible. This subset is denoted with black x markers and a black trend line. This separation of data was done to observe the effect of the cellular geometry separately as the polarization from waxes and hair structures are indistinguishable from the polarization caused by cellular structure. In Fig. 6a the correlation between cell size and *R*_*Qav*_ is low for the full dataset but a slight correlation can be observed for the sub-set of glabrous leaves. This correlation is very weak, but the trend agrees with the expected observation as larger cells result in a higher *R*_*Qav*_. Larger epidermal cells have the potential to create a more optically smooth surface with fewer angular-variant grooves in a given area. Leaves with small epidermal cells are more likely to produce an optically rough surface with large variation in local surface angle due to the constantly undulating cell-groove interface.

In Fig. 6b, no correlation is observed between cell margin undulation and *R*_*Qav*_ for non-glabrous leaves. However, in the glabrous sub-set, a weak correlation between *R*_*Qav*_ and the margin undulation can be observed. This correlation, however, indicates a decrease in *R*_*Qav*_ as the cells become more round (the edge is undulating less). This finding does not fit with the expectation that more undulations would create higher variability in the incident light angle and may relate to the relative heights of undulating and round cells.

The cell cap aspect ratio correlation is shown in Fig. 6c. In previously developed models, an increased epidermal cell cap aspect ratio causes a decrease in reflectance with nadir illumination and has a negligible effect on specular reflectance [20, 34]. In the data collected here, this cellular feature shows no correlation between ${R}_{{Q}_{av}}$ and the cell cap aspect ratio for either the glabrous leaves or the full set of leaves. For all correlations shown in Fig. 6, the largest p-value is 1.37 × 10^–32^.

### Surface waxes

As described in previous research [28] surface waxes can present with different thicknesses and morphologies that affect their appearance at the ultra-microscopic and visible levels. In this work, waxes are classified into two major categories—glossy and glaucous—with the former appearing shiny on the leaf and the latter having a bluish hue like the bloom on a succulent. The differences between these two categories can be seen with the naked eye and in the microscope images and are also distinguishable using spectral measurements.

#### Glossy waxes

**Fig. 7:**
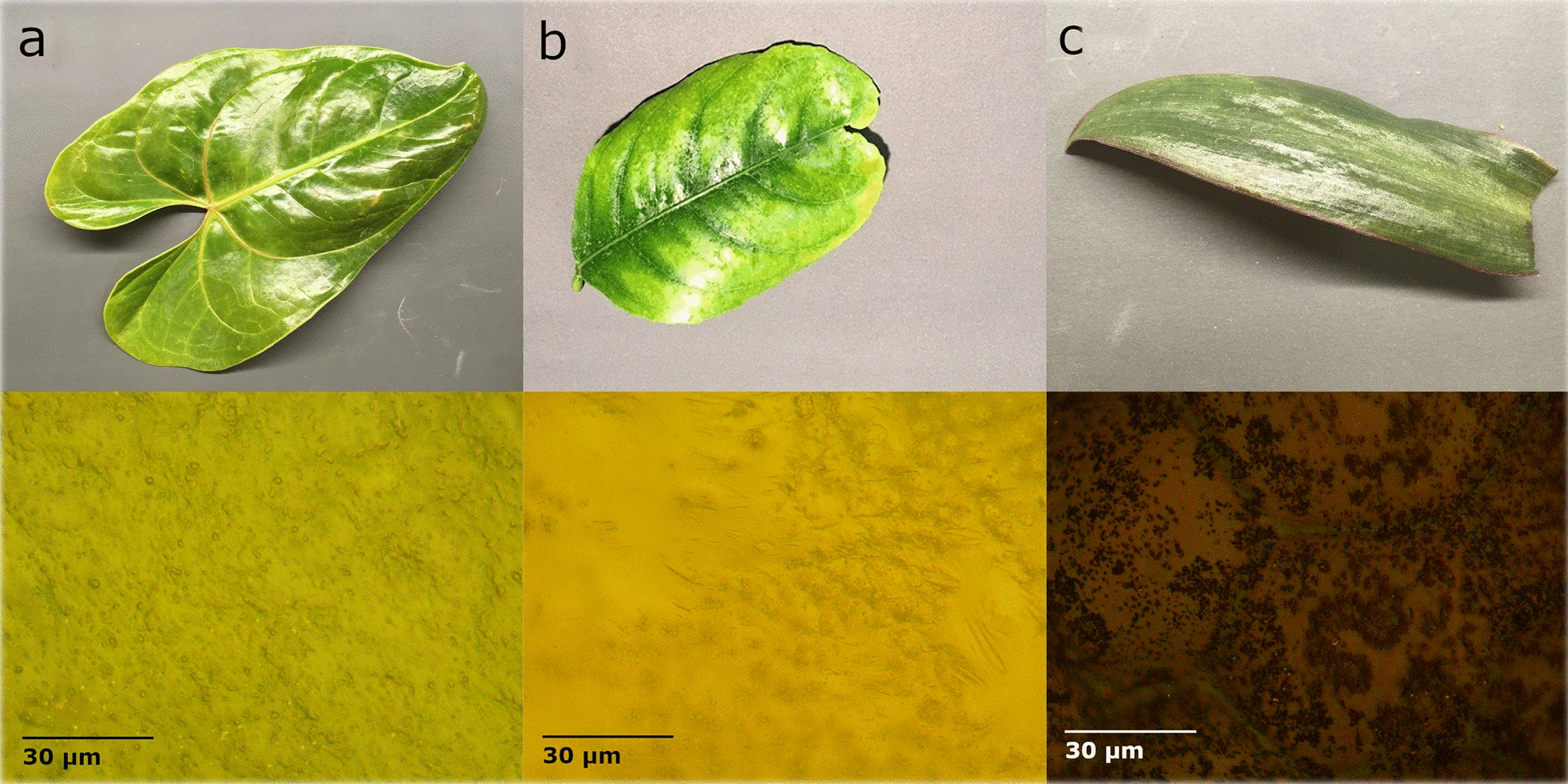
Images of leaves with a glossy, shiny wax **a** Anthurium (*Anthurium* sp. Schott) **b** Lemon (*Citrus limon* (L.) Osbeck) **c** Spiderwort (*Tradescantia* sp. L.). Microscope images at 500 × magnification are shown directly below each leaf sample

To the naked eye, glossy waxes produce a shiny surface on the leaf. When viewed at an angle, these leaves appear to reflect white light. Three examples of leaves with a glossy wax are shown in Fig. 7 with their respective microscope images. Under the microscope, the glossy wax is visible and often covers the epidermal cells completely so that their size and shape cannot be determined. This is the case in the anthurium leaf (Fig. 7a) where the wax layer is producing a smooth film across the entire surface. The wax itself has some texture or what appear to be bubbles within the wax, but this layer is very smooth compared to the roughness of the surface cells. The lemon leaf wax (Fig. 7b) appears to be filling in the space between the epidermal cells to create a smooth surface on portions of the leaf. The spiderwort plant (Fig. 7c) has very large cells which are still visible underneath the surface wax. This wax is covering the whole surface in a thin layer.

The shiny appearance of these leaves results from the more optically smooth surface that is produced when the waxes fill in the grooves between the epidermal cells. The smooth surface allows for consistent directional reflectance of polarized light. Spectrally, this produces more variation between specular and diffuse reflectance, and therefore a higher *R*_*Q*_.

#### Glaucous waxes

**Fig. 8:**
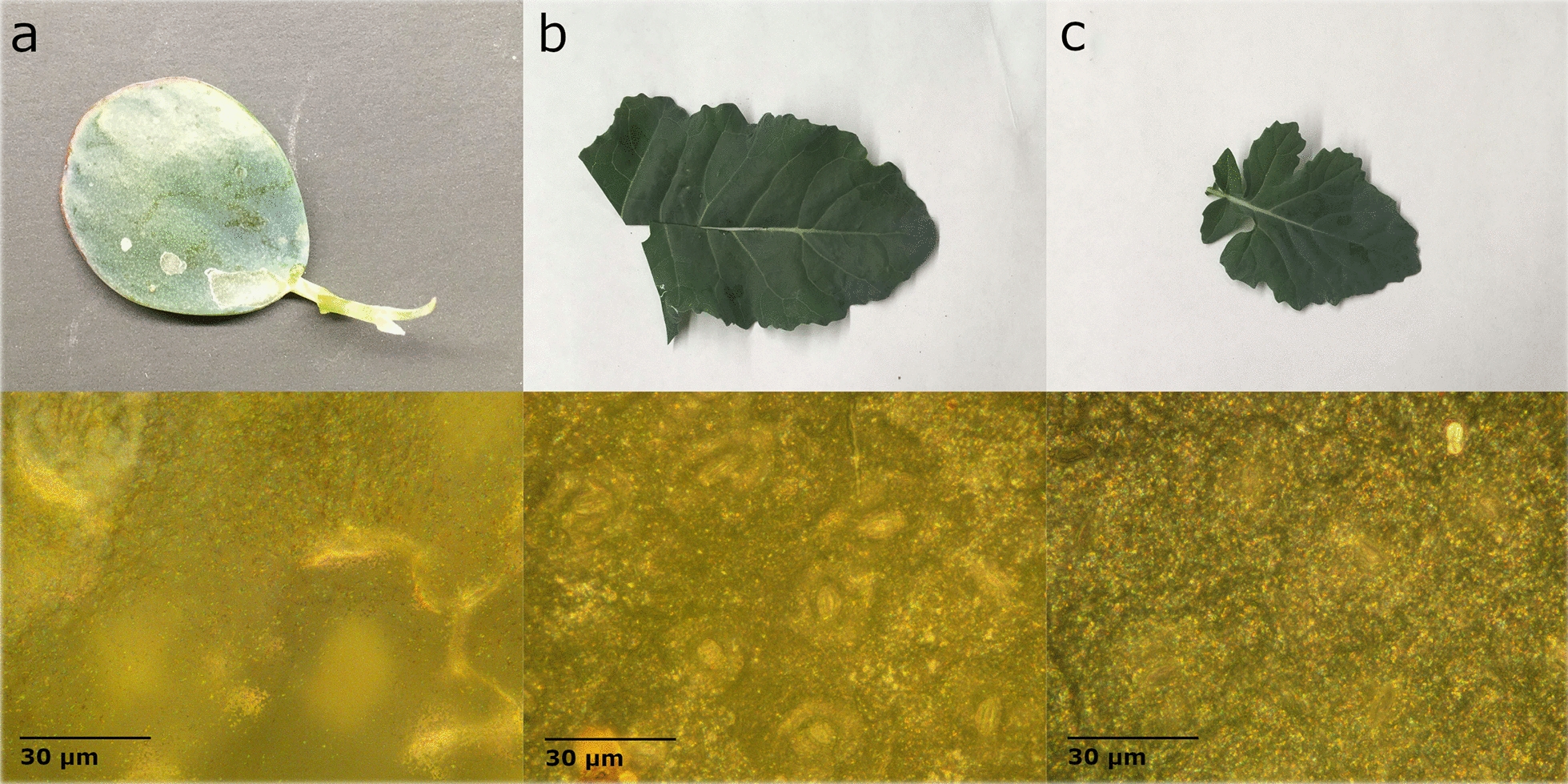
Images of leaves with a glaucous wax **a** Jade (*Crassula ovata* (Miller) Druce (1917)) **b** Broccoli (*Brassica oleracea* L.) **c** Canola (*Brassica napus* L.). Microscope images at 500 × magnification are shown directly below each leaf sample

Glaucous waxes produce a blueish hue on the leaf surface and can often be wiped away to produce a shiny film. This blueish hue is observed as increased spectral reflectance in the visible region. Leaves with this feature were handled very carefully to avoid disturbing the surface. Three leaves with undisturbed surfaces are shown in Fig. 8. In the microscope images, the glaucous leaves can appear as though they are out of focus. The glaucous wax coats the entire surface of the leaf similar to the glossy anthurium, but instead of the textured, bubbled appearance of the glossy wax, the glaucous wax appears more mottled or speckled. Unlike glossy waxes, glaucous waxes form complex structures and scatter light in a less uniform pattern. This results in lower degrees of polarization.

### Trichomes

The shape, size, and density of trichomes affect the reflectance of incident light by creating variable surface heights and angles resulting in non-uniform specular reflectance. In this section, trichomes are separated into glandular and non-glandular with the latter being much more prevalent in the leaves presented in this study.

#### Glandular trichomes

Glandular trichomes are hair like structures on the surface of the leaf that secrete metabolites [27]. Of the leaves studied in this work, glandular trichomes were only identified on two tomato leaves. When comparing the spectral measurements of the leaves with glandular trichomes to those with non-glandular trichomes, no distinguishable differences were found. The presence of the secretions on the tips of the trichomes does not appear to affect the surface polarization in a capacity that is different to the non-glandular trichomes.

#### Non-glandular trichomes

Non-glandular trichomes were found on 39 of the leaves studied in this work (including two which displayed both glandular and non-glandular trichomes). To quantify the percentage of the surface that was covered with pubescence, hairs on each leaf were manually traced. The percentage of the surface covered by the traced hairs was taken as the percent pubescent coverage. For leaves with high pubescent coverage (greater than 25%), and small but consistent sized trichomes, an approximation was determined by manually-counting the number of hairs and multiplying this value by the average size of 5 individual hairs.

**Fig. 9:**
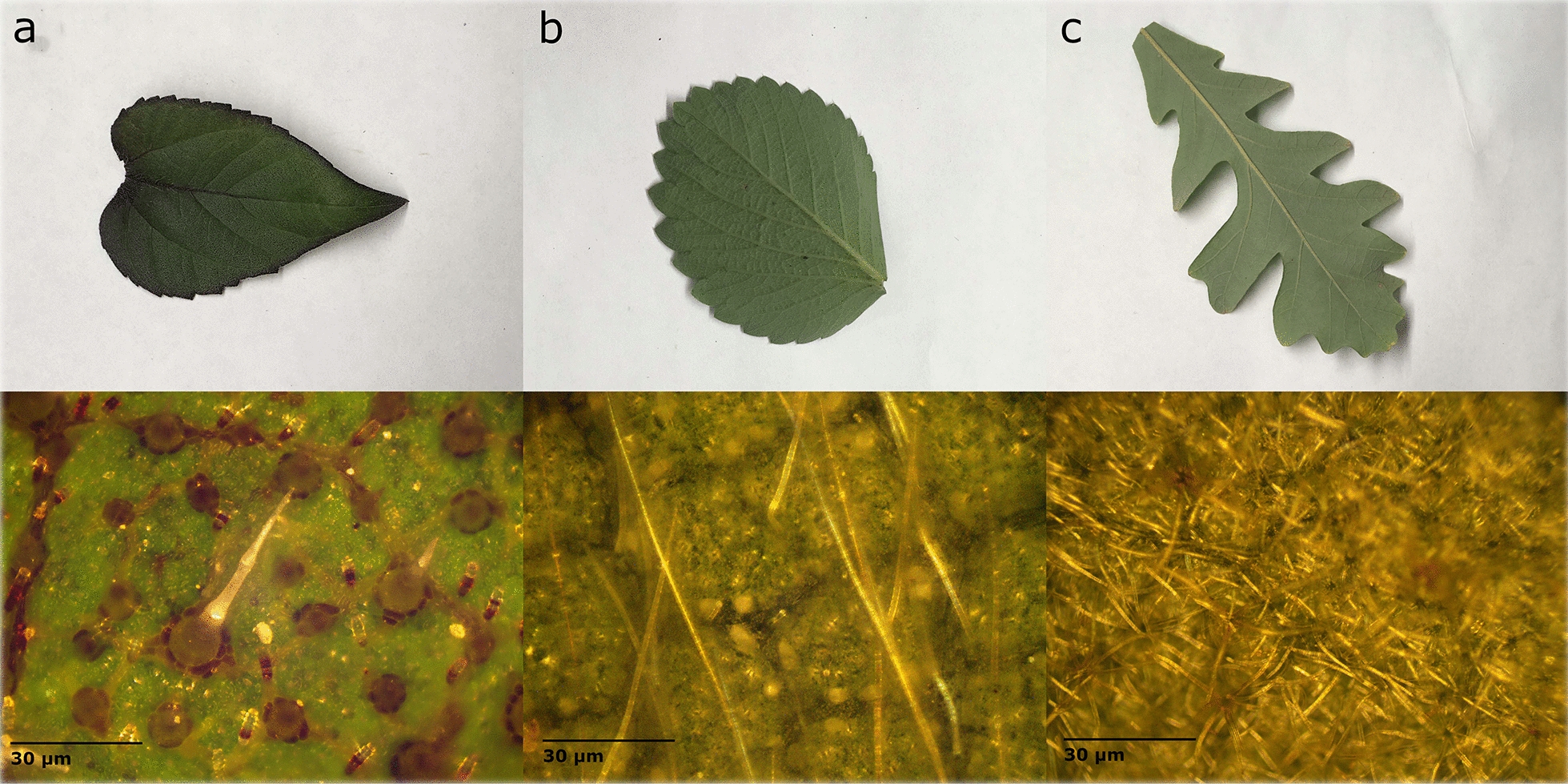
Image of leaves with different types of non-glandular trichomes **a** Sunflower (*Helianthus annuus* L.) **b** Strawberry (*Fragaria x ananassa* Duchesne) **c** Oak (*Quercus* sp. L.). Microscope images at 100 × magnification are shown directly below each leaf sample

**Fig. 10:**
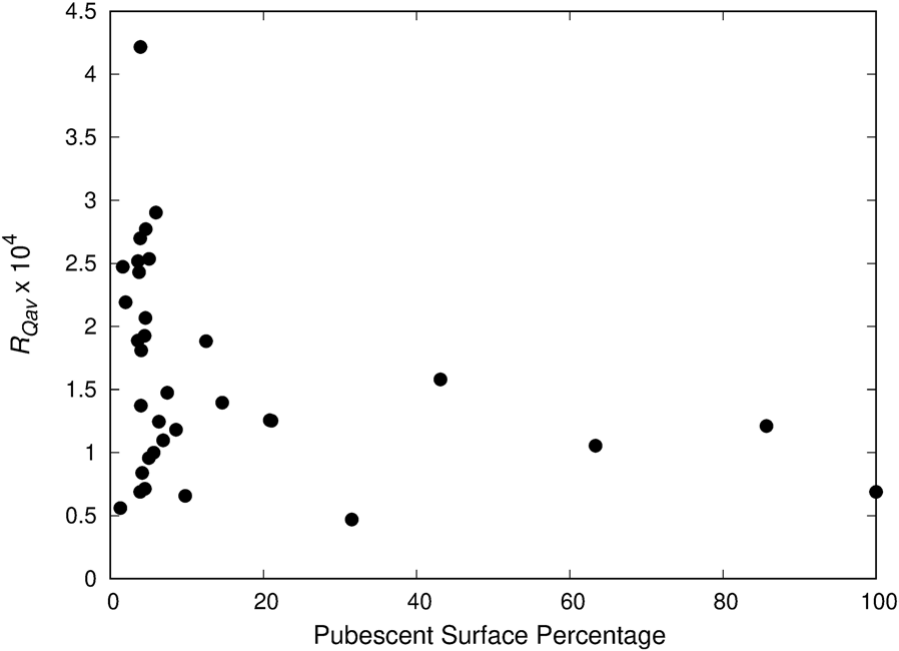
The effect of pubescence on *R*_*Qav*_ value based on surface area percentage covered with trichomes

Within the non-glandular classification, trichomes of different sizes, shapes, and dispersion densities were found as shown in Fig. 9. When the trichome size and density are high, the *R*_*Qav*_ value is very low as the light is scattered more randomly on the trichomes. However, as seen in Fig. 10, the relationship between the pubescence covering the surface and *R*_*Qav*_ is not linear. The size and shape of the trichome can also affect the scattering of light. For medium density (less than 10% of the surface covered) of primarily short vertical trichomes less than 20 μm in length (as in Fig. 9a) *R*_*Qav*_ is measured between 0.5 × 10^–4^ and 4.5 × 10^–4^. The sunflower shown in Fig. 9a has an *R*_*Qav*_ of 1.92 × 10^–4^. The long, horizontal trichomes on the strawberry in Fig. 9b result in a lower *R*_*Qav*_ of 1.18 × 10^–4^. Although both these leaves have pubescent surface coverage under 10% (4.5% for sunflower and 8.6% for strawberry) the horizontal trichomes may scatter the light more effectively, lowering the *R*_*Qav*_. For leaves with extreme pubescence, the *R*_*Qav*_ value is very low (under 2 × 10^–4^) and *DIFF*_*R*_ decreases as well. For these high trichome density leaves, the trichomes are so numerous that a nearly opaque layer of trichomes can be seen above the epidermal layer. The oak shown in Fig. 9c has trichomes covering 63.4% of the surface and an *R*_*Qav*_ of 1.05 × 10^–4^. These highly pubescent leaves often appear blueish or whitish similar to the glaucous leaves and have a comparable *DIFF*_*R*_.

### Leaf surface phenotype classification

The effects of leaf surface phenotypes have been discussed generally in relation to spectral measurements, but the overlapping effects of surface roughness, waxes, and trichomes makes surface phenotype classification non-trivial. The effects of cellular surface roughness are most evident when there is little to no wax or hairs present on the leaves. In general, the effects of hairs and waxes on *R*_*Qav*_ are more prominent than the cellular effects. This can be observed in Fig. 6 where the glabrous leaves have *R*_*Qav*_ values ranging from 0.48 × 10^–4^ to 4.32 × 10^–4^ but the hairy and waxy leaves have *R*_*Qav*_ values ranging from 0.30 × 10^–4^ to 19.63 × 10^–4^. These stronger effects on *R*_*Qav*_ provide a more clear method for identifying these phenotypes.

**Fig. 11:**
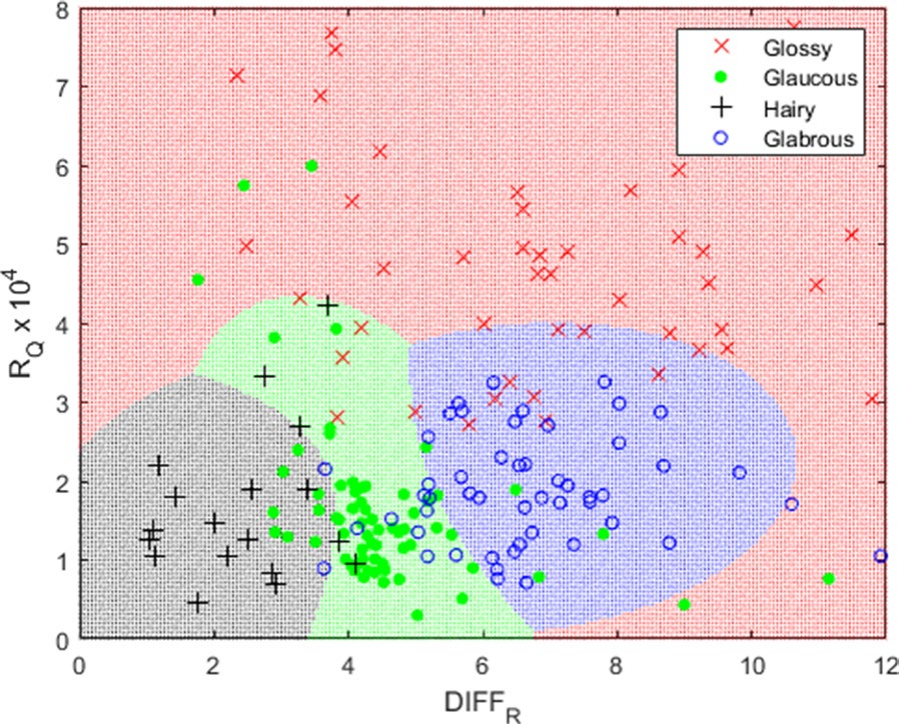
Classification space of leaf surface phenotypes based on *R*_*Qav*_ and *DIFF*_*R*_ for one of 10,000 runs (classification rate was 74.9% in this example)

To compare the effects of these larger-scale phenotypes, four categories of classification were developed (with number of samples shown in parenthesis): glossy (waxy) (104), glaucous (110), hairy (39), and glabrous (defined here as leaves without significant trichomes or waxes) (96). Using *R*_*Qav*_ and *DIFF*_*R*_, a quadratic discrimination analysis with equal prior probabilities was performed to classify the samples. For one example using 150 training samples selected randomly (50 glossy, 48 glaucous, 18 hairy, and 34 glabrous) and 199 testing samples (54 glossy, 62 glaucous, 21 hairy, and 62 glabrous), the leaf surface phenotypes were correctly identified for 74.9% of samples. These results in the classification space are shown in Fig. 11 and resulted in correct identification for 78.1% of glossy, 72.7% of glaucous, 72.2% of hairy, and 74.5% of glabrous samples. The portion of the classification space for *R*_*Qav*_ values from 8 × 10^–4^ to 20 × 10^–4^ were not included as they only contained samples classified as glossy and crowded the visualization of the portion of the graph where phenotype classification spaces are presented.

**Fig. 12:**
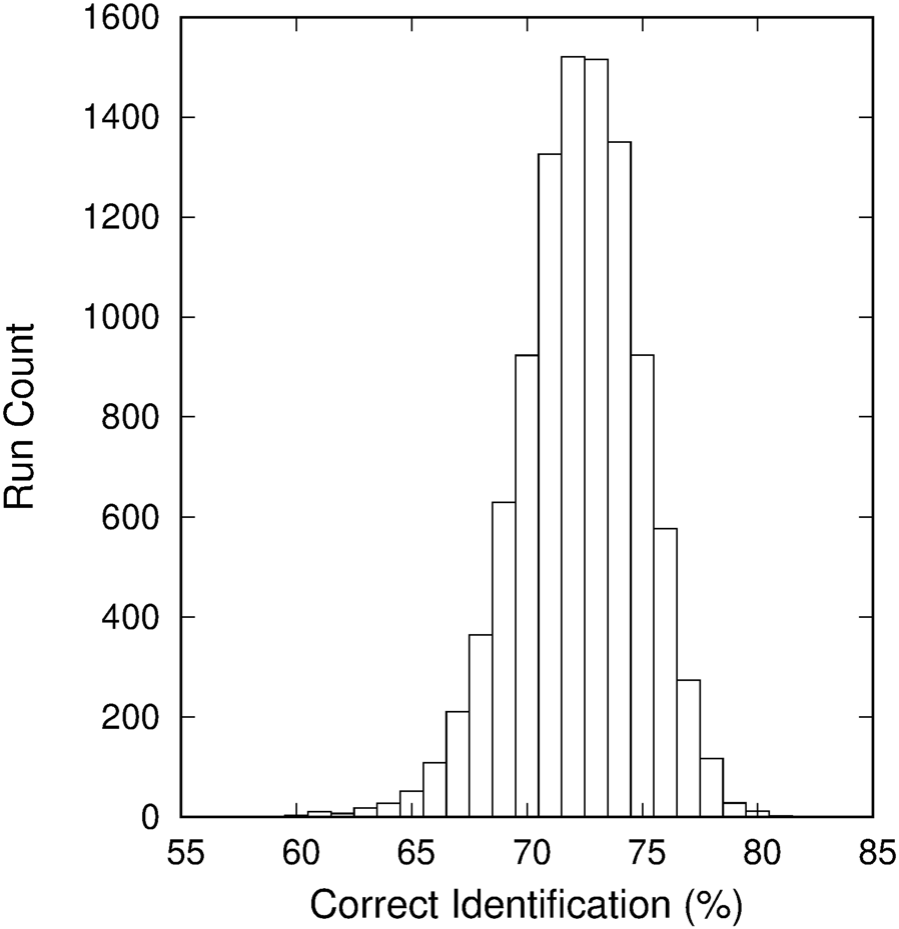
Classification distribution results for 10,000 runs with random data splitting

To more accurately test the ability to classify the surface phenotypes, the data were randomly split again into a new training (150 samples) and testing (199 samples) group and a new classification rate was determined. This process was repeated for 10,000 runs and the aggregate results were used for classification analysis. The average correct classification rate was 72.9% with the worst and best runs finding a correct classification rate of 60.3% and 81.4%, respectively. The standard deviation for the average classification rate was 2.7%. The results of all 10,000 runs are summarized in Fig. 12 showing the distribution of classification rates.

**Fig. 13:**
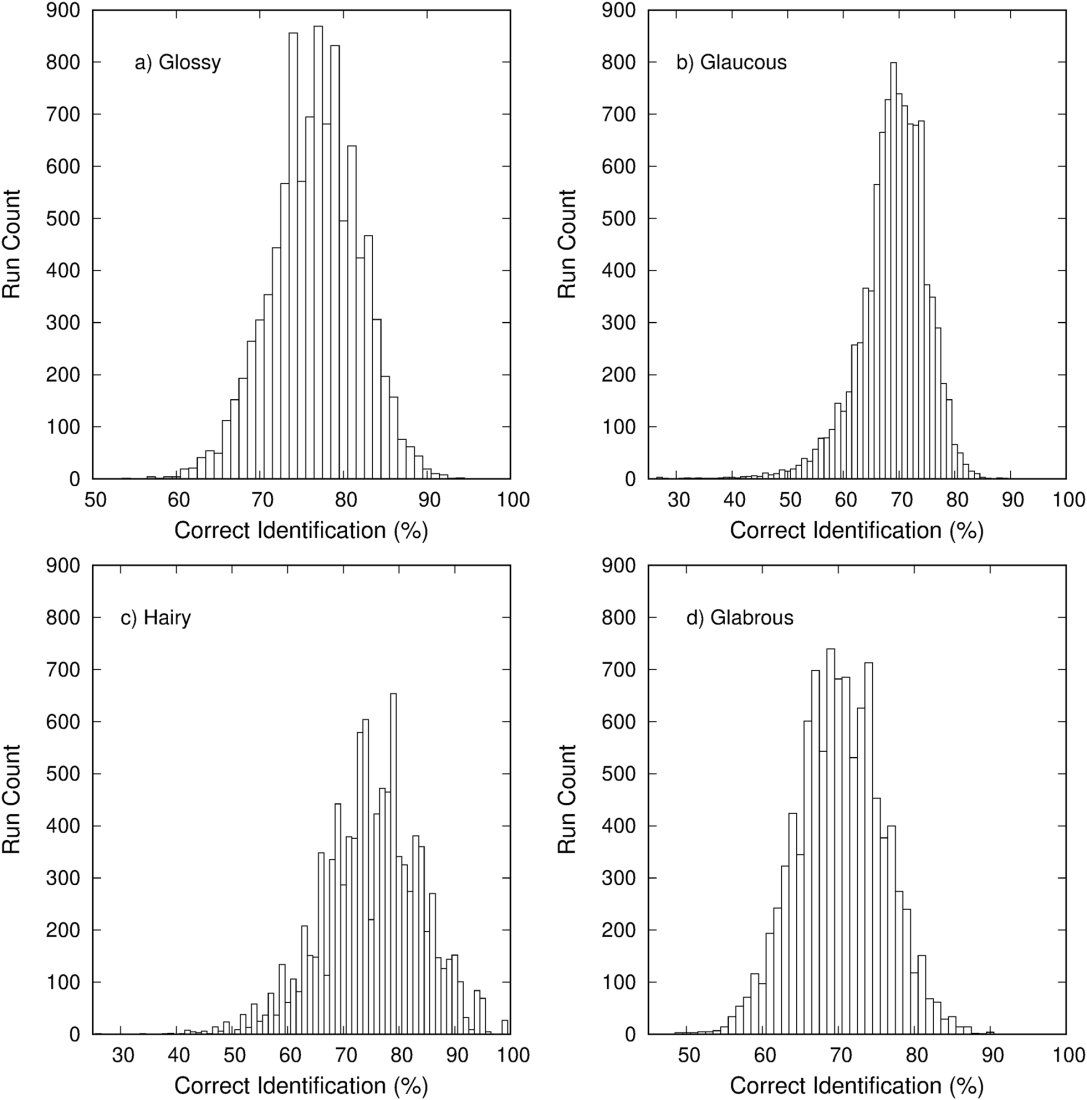
Classification distribution results for **a** glossy, **b** glaucous, **c** hairy, and **d** glabrous leaves

The average correct classification rates (with standard deviations) for each surface phenotype were 77.2% $\pm$ 5.2% for glossy (12a), 69.5% $\pm$ 6.1% for glaucous (12b), 75.8% $\pm$ 8.9% for hairy (12c), and 70.8% $\pm$ 5.7% for glabrous (12d). The distributions for the classifications rates for each surface phenotype are shown in Fig. 13. These results indicate that differentiating between surface phenotypes is possible using spectral measurements combined with a polarizing filter.

In this study, a variety of leaf colors and ages were investigated including light green to dark red leaf samples. However, it was noted that for samples with very low chlorophyll content (pink, yellow, or white leaves), the diffuse ratio wavelengths would need to be adjusted. Identifying these leaves is trivial using spectral measurements and such leaves have been successfully classified using a lower visible wavelength band (results not shown).

Although quantifying the wax loading or trichome characteristics were not considered in this study, future work should consider the potential quantitative application of these methods. The results from this study have produced a classification system that can use polarized light reflected at Brewster’s Angle to determine leaf surface phenotypes at the macro and microscopic levels. Further research will also investigate the effects of illumination at angles other than Brewster’s to improve the robustness of this classification for use with more generalized measurements. These classifications will help in remote sensing and precision agriculture by improving the specificity of leaf modelling methods.

## Conclusions

Three hundred forty nine leaf samples were analyzed microscopically and spectrally to determine the feasibility of characterizing the leaf surface phenotype using spectral measurements. Microscope images were used to determine the cell size, the degree of undulation of the cell edge, and the cell cap aspect ratio. The presence of hairs and waxes were also analyzed under the microscope. These data along with visual examination of the leaves were used to classify leaves into four categories: glossy wax, glaucous wax, high trichome density, and glabrous (low wax or trichome loading). Using these four categories and by splitting the data into training and testing sets, a discriminant analysis with a quadratic function was repeated for 10,000 iterations resulting in an average classification rate of 72.9% and a standard deviation of 2.7%. The effects of microscopic surface features such as cell size and cell cap aspect ratio were not found to have a significant correlation with the spectral measurements obtained in this study. These measurements may still be useful in leaf optically modeling through their incorporation into existing models. Future work will investigate the potential for quantitatively assessing wax and trichome loading as well as the effects of angular variation.

## Data Availability

The datasets obtained and/or analyzed during the current study are available in part through the published LOTUS dataset [32]. Additional samples are available from the corresponding author on request.
